# A decade-long assessment reveals persistent vitamin D deficiency and its associated factors in early-pregnant women from northern China (2016–2025)

**DOI:** 10.3389/fnut.2026.1881959

**Published:** 2026-08-25

**Authors:** Haizhen Chen, Quan Yun, Xitian Fan, Chunli Xu, Jingjing Wei

**Affiliations:** 1 Laboratory Department, Shanxi Provincial Children’s Hospital, Taiyuan, Shanxi, China; 2 Department of Pediatrics, Shanxi Medical University, Taiyuan, Shanxi, China; 3 Department of Pediatric Intensive Care Unit, Shanxi Provincial Children’s Hospital, Taiyuan, Shanxi, China

**Keywords:** cohort study, early pregnancy, northern China, seasonal variation, vitamin D

## Abstract

**Background:**

Large-scale data on vitamin D status in early-pregnant women in high-risk Northern China are scarce. This study evaluated the decadal trends (2016–2025) and associated factors of serum 25(OH)D levels in this population in Shanxi Province.

**Methods:**

This retrospective cohort included 20,693 early-pregnant women (<14 weeks). Serum 25(OH)D was measured. Logistic regression assessed associations with of season, age, conception mode, and the COVID-19 period.

**Results:**

The median 25(OH)D was 36.12 nmol/L, indicating a concerning status. Season was the strongest associated factor. The pandemic period (2020–2022) saw a significant rise in severe deficiency (<30 nmol/L) to 35.6%. Advanced maternal age (≥35) and ART conception were linked to higher levels. Summer and autumn were protective factors for sufficiency, while natural conception was an associated factor for deficiency.

**Conclusion:**

Severe vitamin D deficiency is widespread. Findings advocate for routine perinatal vitamin D screening and supplementation, with tailored strategies for winter and specific subgroups.

## 1 Introduction

Vitamin D is a classic vitamin involved in calcium and phosphorus metabolism, essential for maintaining skeletal health (1). Concurrently, it plays significant roles in gene regulation, cell proliferation and differentiation, immune regulation, and inflammation suppression (2–4). However, vitamin D deficiency or insufficiency has evolved into a severe global public health challenge, closely associated with an increased risk of osteoporosis, autoimmune diseases, cardiovascular metabolic diseases, and certain malignancies (5). This issue is particularly prominent among high-risk populations (e.g., pregnant women, children, and the elderly) (5–7).

Pregnant women are one of the most critical groups for nutritional intervention. Substantial evidence indicates that maternal vitamin D deficiency is closely associated with adverse pregnancy outcomes (8, 9) and is also an important factor associated with the future growth and development of the offspring (10–14). Based on the Developmental Origins of Health and Disease (DOHaD) hypothesis, the nutritional and environmental conditions experienced by the fetus in utero are directly linked to intrauterine growth and future disease risk (15, 16).

Despite the consensus on the importance of vitamin D for maternal and infant health, baseline levels and their dynamic characteristics vary significantly across different regions and populations (17). Shanxi Province is located in the mid-to-high latitude region of Northern China (34°34′–40°44′N), characterized by long winters with weak sunlight intensity and short duration. Residents theoretically belong to a relatively high-risk group for vitamin D deficiency, as they often cannot rely on endogenous synthesis for a considerable part of the year (18). However, to date, large-scale, long-term studies on the vitamin D nutritional status of early-pregnant women in Shanxi Province are still lacking.

The first trimester is the most critical period for fetal organ development (14, 19). Furthermore, detecting vitamin D deficiency or insufficiency in early pregnancy allows for timely supplementation tailored to individual circumstances. Therefore, this study aims, through a large-scale, single-center, 10 year retrospective study (2016–2025), to systematically reveal the true distribution status and decadal trends of vitamin D levels in early-pregnant women in Shanxi Province, and to deeply analyze the influence of various factors such as season, maternal age, number of fetuses, study periods, and conception mode on these levels. This will fill an important geographical research gap and provide the highest level of evidence-based medical support for implementing targeted vitamin D screening and supplementation strategies in this region.

## 2 Materials and methods

### 2.1 Study population

This retrospective study analyzed data from healthy pregnant women who underwent Vitamin D examination at Shanxi Maternal and Child Health Hospital between January 2016 and December 2025. *Inclusion criteria*: (1) Pregnant women aged 15–49 years; (2) No family history of hypertension, diabetes, heart disease, etc.; (3) Pregnant female, previously healthy; (4) No adverse pregnancy history. *Exclusion criteria*: (1) Gestational age ≤1 or ≥14 weeks; (2) Pregnancy complications, such as hypertensive disorders of pregnancy, gestational diabetes mellitus, etc.; (3) Comorbidities like obesity, anemia, hyperthyroidism, hypothyroidism; (4) Threatened abortion; (5) Threatened preterm labor; (6) Missing data. The participant selection process is detailed in the flow diagram (Supplementary Figure S1). All included subjects provided informed consent. This study complied with the ethical requirements of Shanxi Maternal and Child Health Hospital (IRB-KYYN-2026-G005).

### 2.2 Data collection

All clinical data were retrospectively extracted from the hospital’s electronic medical record system (EMRS) by two independent researchers using a standardized data abstraction form. The abstracted variables included blood collection year, season, serum 25(OH)D level, maternal age, number of fetuses, parity, conception mode, and gestational age at blood draw. Blood collection years spanned from 2016 to 2025. Discrepancies in data extraction were resolved through discussion with a third senior researcher. Non-clinical data—such as season definition based on local meteorological records and period classification according to previous other studies (20, 21). Based on the climatic characteristics of Shanxi Province, seasons were defined as Spring (March–May), Summer (June–August), Autumn (September–November), and Winter (December–February) (22).

The study period was divided into three phases: pre-pandemic, during pandemic, and post-pandemic. Vitamin D status was categorized as severe deficiency (<30 nmol/L), deficiency (≥30 and <50 nmol/L), insufficiency (≥50 and <75 nmol/L), and sufficiency (≥75 nmol/L) (17, 21–26). Pregnancy type was classified as adolescent pregnancy (<20 years) (23), appropriate-age pregnancy (20–34 years), and advanced maternal age (AMA) pregnancy (≥35 years). Number of fetuses was classified as singleton, twin, or triplet; Number of fetuses as primipara or multipara; and conception mode as natural conception or *in vitro* fertilization-embryo transfer (IVF-ET).

To ensure clarity in reporting, we defined the following composite terms: ① Total deficiency: The sum of severe deficiency (<30 nmol/L) and deficiency (≥30 and <50 nmol/L). It was calculated as (Number of women with severe deficiency + Number of women with deficiency)/Total number of women × 100%. ② Combined rate of deficiency and insufficiency: The sum of severe deficiency (<30 nmol/L), deficiency (≥30 and <50 nmol/L), and insufficiency (≥50 and <75 nmol/L). It was calculated as (Number of women with severe deficiency + Number of women with deficiency + Number of women with insufficiency)/Total number of women × 100%.

### 2.3 Sample collection and 25(OH)D measurement

Venous blood was collected into vacuum tubes (BD Vacutainer, 5 mL capacity). Serum samples were immediately separated by centrifugation at 3500 rpm, 4 °C for 10 min and aliquoted for storage at −80 °C. Serum concentration of 25-hydroxyvitamin D was measured using electrochemiluminescence method according to the protocal of Elecsys Vitamin D total III detection kit provided by the manufacturer. The testing instrument is the cobas® e 601 module fully automatic analyzer (Roche, United States). According to the manufacturer’s specifications, the measuring range is 7.50–300 nmol/L (3.00–120 ng/mL); samples exceeding the upper limit are automatically diluted and reanalyzed. Calibration is performed using PreciControl Vitamin D total III calibrators traceable to internal Roche standards. Intra-assay coefficients of variation (CVs) are <5% and inter-assay CVs are <7% across the physiological range (20–60 ng/mL), as validated per CLSI EP05-A3 guidelines.

### 2.4 Statistical analysis

Statistical analysis was performed using GraphPad Prism 8.0 software. Measurement data conforming to normal or approximately normal distribution were expressed as mean ± standard deviation (SD). Comparisons between two groups with homogeneity of variance used the *t*-test, while comparisons among multiple groups used one-way ANOVA. For data with heterogeneous variance, Welch’s corrected *t*-test or the Kruskal-Wallis rank sum test was used, respectively. For severely skewed distributions, data were expressed as median (interquartile range, IQR) and analyzed using non-parametric tests. Count data were expressed as number and percentage (%), and group comparisons were made using the Chi-square test. Univariate and multivariate logistic regression analyses were employed to assess associated factors.

Covariates included in the multivariable regression models were selected *a priori* based on established evidence from prior epidemiological literature, rather than via data-driven approaches, to minimize the risk of selective reporting. Specifically, we adjusted for maternal age, season of blood collection, conception mode (natural conception vs. ART), period, and number of fetuses, as these factors have been consistently identified as significant correlates of vitamin D status in pregnant populations (22, 24–34).

To examine the robustness of our primary findings, sensitivity analyses were performed. First, we excluded participants aged <18 years and those with triplet pregnancies, then repeated the multinomial logistic regression model using identical specifications. Second, serum 25(OH)D was treated as a continuous outcome. Due to its right-skewed distribution, a log10 transformation (lg10_25OHD) was applied, and multiple linear regression was performed with the same set of covariates as the primary model. Model assumptions were evaluated using residual diagnostics (histogram, P–P plot, scatterplot of standardized residuals vs. predicted values) and collinearity diagnostics (variance inflation factor, VIF).

## 3 Results

### 3.1 Study population characteristics

A total of 20,693 early-pregnant women were included in this study, as shown in the participant flow diagram (Supplementary Figure S1). The median age was 30 years (IQR: 26–33 years), with the majority (84.6%) aged 20–34 years (appropriate-age pregnancy). Most participants had singleton pregnancies (98.9%), were primiparous (99.4%), and conceived naturally (99.3%). The overall median serum 25(OH)D level was 36.12 nmol/L (IQR: 27.87–47.42 nmol/L), with 78.7% of women having total deficiency and only 3.7% achieving sufficiency. Detailed baseline characteristics are presented in Table 1.

**Table 1:** Overview of baseline characteristics.

Total, *n* (%)			20,693 (100)
Age (years), median (IQR)			30 (26–33)
Serum 25(OH)D (nmol/L), median (IQR)			36.12 (27.87–47.42)
Vitamin D status, *n* (%)			
Severe deficiency (<30 nmol/L)			6,504 (31.4)
Deficiency (30–<50 nmol/L)			9,779 (47.3)
Insufficiency (50–<75 nmol/L)			3,637 (17.6)
Sufficiency (≥75 nmol/L)			773 (3.7)
Age group, *n* (%)			
<35 y	17,527 (84.7)	<20 y (adolescent pregnancy)	22 (0.1)
		20–34 y (appropriate age pregnancy)	17,505 (84.6)
≥35 y (advanced age pregnancy)			3,166 (15.3)
Number of fetuses, *n* (%)			
Singleton			20,459 (98.87)
Twin			231 (1.12)
Triplet			3 (0.01)
Parity, *n* (%)			
Primipara			20,579 (99.4)
Multipara			114 (0.6)
Conception mode, *n* (%)			
Natural conception			20,547 (99.3)
IVF-ET			146 (0.7)
Season of blood collection, *n* (%)			
Spring (March–May)			5,608 (27.1)
Summer (June–August)			5,290 (25.6)
Autumn (September–November)			4,900 (23.7)
Winter (December–February)			4,895 (23.6)
Study period, *n* (%)			
Pre-pandemic (2016–2019)			3,381 (16.3)
During pandemic (2020–2022)			8,491 (41.0)
Post-pandemic (2023–2025)			8,821 (42.6)

### 3.2 Distribution by year

**Figure 1:**
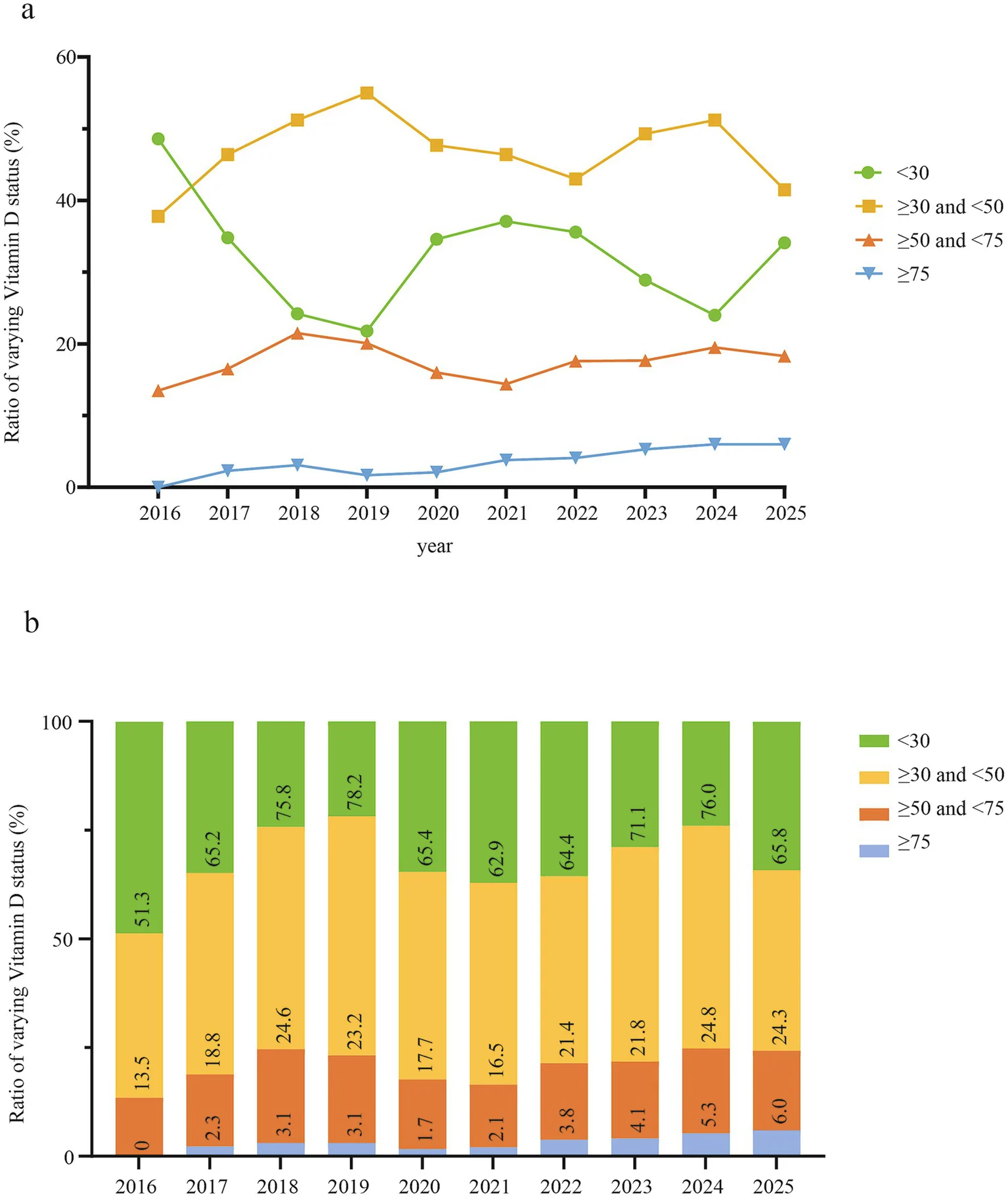
**(a)** Annual trends in the proportion different vitamin D status from 2016 to 2025. **(b)** The annual median serum 25(OH)D levels (nmol/L) from 2016 to 2025.

The annual median vitamin D levels ranged from 30.8 nmol/L (2016) to 38.92 nmol/L (2018) (Supplementary Table S1). Regarding the longitudinal comparison showed that the proportion with severe deficiency exhibited a decreasing trend from 2016 to 2019 (from 35.5 to 21.8%), sharply increased in 2020 (to 34.2%), remained high until 2022 (35.6%), showed a clear decreasing trend starting in 2023, but rose again to nearly 35% in 2025. Throughout the decade, the total deficiency rate consistently exceeded 70%, and the combined rate (<75 nmol/L) remained above 95% in most years. The proportion with sufficiency slightly decreased in 2019 but showed an overall increasing trend over the 10 years. The proportion with insufficiency peaked in 2018 at 21.5%. (Figure 1a) Besides, the cross-sectional distribution of vitamin D status, 31.4% of women had severe deficiency, 47.3% had deficiency, yielding a total deficiency rate of 78.7%. Only 17.6% had insufficiency, and 3.7% achieved sufficiency. When combining deficiency and insufficiency, 96.3% of women had vitamin D levels below 75 nmol/L. (Figure 1b).

### 3.3 Distribution by season

Overall vitamin D levels exhibited a characteristic pattern of being higher in summer and autumn and lower in spring and winter. As shown in Table 2, the serum vitamin D level in summer was 40.23 (31.39, 52.00) nmol/L, in autumn 37.91 (29.36, 49.68) nmol/L, while levels in spring and winter were significantly lower, at 33.17 (25.66, 43.49) nmol/L and 33.46 (26.37, 43.74) nmol/L, respectively.

**Table 2:** Serum 25(OH)D levels across seasons, pregnancy status, maternal age, periods, parity, number of fetuses, and conception modes.

Variable	Vitamin D (nmol/L)
Season	
Spring	33.17 (25.66, 43.49)
Summer	40.23 (31.39, 52.00)
Autumn	37.91 (29.36, 49.68)
Winter	33.46 (26.37, 43.74)
Pregnancy status	
Adolescent pregnancy (<20 y)	37.07 (24.92, 43.55)
Appropriate age pregnancy (20–34 y)	35.80 (27.63, 46.83)
Advanced age pregnancy (≥35 y)	38.21 (29.23, 50.35)
Maternal age	
<35	35.80 (27.63, 46.83)
≥35	38.21 (29.23, 50.35)
Period	
Pre-pandemic (2016–2019)	37.64 (29.48, 48.34)
During pandemic (2020–2022)	34.50 (26.61, 45.24)
Post-pandemic (2023–2025)	36.98 (28.65, 49.14)
Parity	
Primipara	36.12 (27.87, 47.41)
Multipara	38.53 (29.87, 50.04)
Number of fetuses	
Singleton	36.11 (27.86, 47.36)
Twins	37.50 (28.60, 51.89)
Triplets	55.06 (46.46, 62.72)
Mode of conception	
Natural conception	36.10 (27.87, 47.38)
IVF-ET	39.42 (29.61, 52.98)

**Figure 2:**
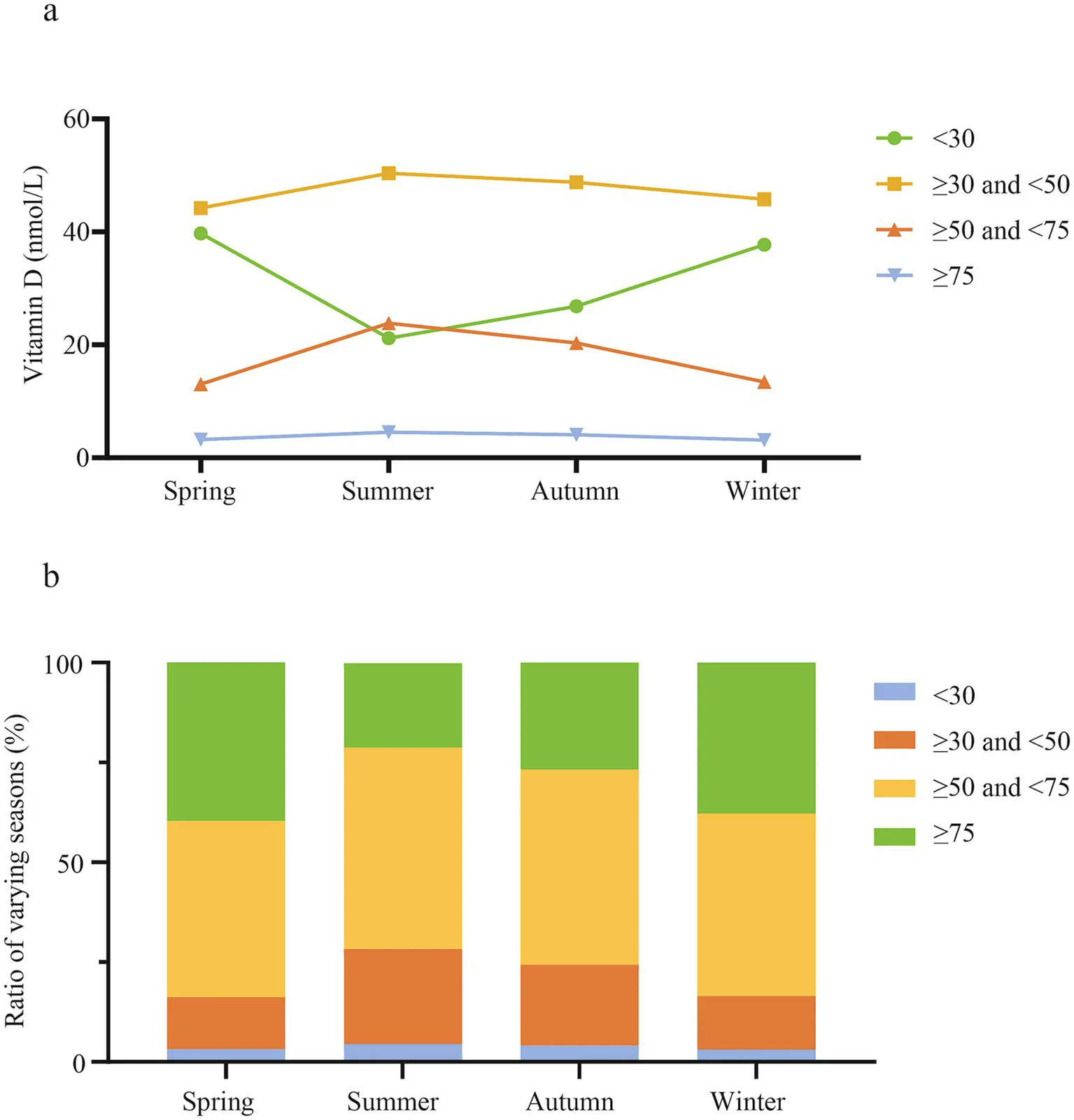
Seasonal variation in vitamin D status. **(a)** The median serum 25(OH)D levels (nmol/L) across seasons. **(b)** The percentage of participants within different vitamin D status according to season.

**Figure 3:**
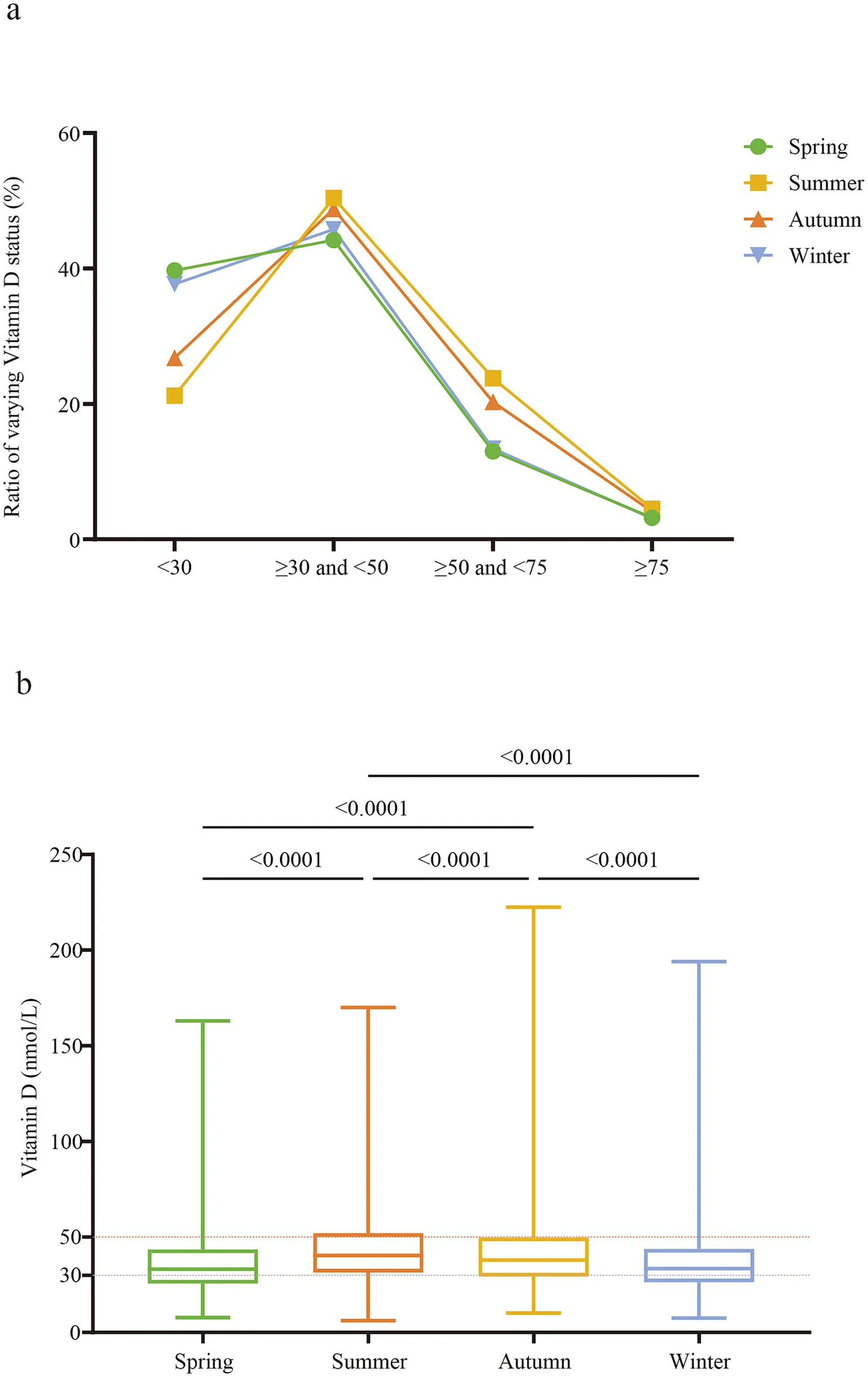
**(a)** The proportion of vitamin D sufficiency and severe deficiency across different seasons. **(b)** The distribution of serum 25(OH)D concentrations across four seasons.

The severe deficiency rate was lowest in summer (21.2%) and highest in spring (39.7%) and winter (37.7%). The deficiency rate was relatively stable across seasons (44.3–48.4%). Consequently, the total deficiency rate was highest in spring (88.1%) and winter (82.0%), and lowest in summer (65.5%). The insufficiency rate peaked in summer (23.8%) and autumn (20.3%), and was lowest in winter (14.9%). The sufficiency rate was highest in summer (4.5%) and lowest in winter (3.1%). The combined rate was 95.5% in summer, 95.9% in autumn, 96.9% in winter, and 97.8% in spring (Figures 2a,b, 3a and Table 3).

**Table 3:** Distribution of vitamin D status across seasons, pregnancy status, maternal age, periods, number of fetuses, and conception modes.

Variable	Vitamin D status *n* (%)			
	Sufficiency	Insufficiency	Deficiency	Severe deficiency
Season				
Spring	179 (3.2%)	728 (13.0%)	2,477 (44.2%)	2,224 (39.7%)
Summer	240 (4.5%)	1,259 (23.8%)	2,667 (50.4%)	1,124 (21.2%)
Autumn	202 (4.1%)	996 (20.3%)	2,391 (48.8%)	1,311 (26.8%)
Winter	150 (3.1%)	656 (13.4%)	2,244 (45.8%)	1845 (37.7%)
Pregnancy status				
Adolescent pregnancy (<20 y)	0 (0.0%)	3 (13.6%)	12 (54.5%)	7 (31.8%)
Appropriate age pregnancy (20–34 y)	626 (3.6%)	2,968 (17.0%)	8,262 (47.2%)	5,649 (32.3%)
Advanced age pregnancy (≥35 y)	145 (4.6%)	668 (21.1%)	1,505 (47.5%)	848 (26.8%)
Maternal age				
<35	626 (3.6%)	2,971 (17.0%)	8,274 (47.2%)	5,656 (32.3%)
≥35	145 (4.6%)	668 (21.1%)	1,505 (47.5%)	848 (26.8%)
Period				
Pre-pandemic (2016–2019)	96 (2.8%)	660 (19.5%)	1731 (51.2%)	894 (26.4%)
During pandemic (2020–2022)	223 (2.6%)	1,344 (15.8%)	3,857 (45.4%)	3,067 (36.1%)
Post-pandemic (2023–2025)	452 (5.1%)	1,635 (18.5%)	4,191 (47.5%)	2,543 (28.8%)
Parity				
Primipara	768 (3.7%)	3,613 (17.6%)	9,723 (47.2%)	6,475 (31.5%)
Multipara	3 (2.6%)	26 (22.8%)	56 (49.1%)	29 (25.4%)
Number of fetuses				
Singleton	758 (3.7%)	3,584 (17.5%)	9,675 (47.3%)	6,442 (31.5%)
Twins	13 (5.6%)	53 (22.9%)	103 (44.6%)	62 (26.8%)
Triplets	0 (0.0%)	2 (66.7%)	1 (33.3%)	0 (0.0%)
Mode of conception				
Natural conception	758 (3.7%)	3,609 (17.6%)	9,713 (47.3%)	6,467 (31.5%)
IVF-ET	13 (8.9%)	30 (20.5%)	66 (45.2%)	37 (25.3%)

Box plots (Figure 3b) further confirmed this distribution difference, showing that the median and interquartile range of vitamin D concentrations in summer were significantly higher than in other seasons, with statistically significant differences between groups.

### 3.4 Maternal age

**Figure 4:**
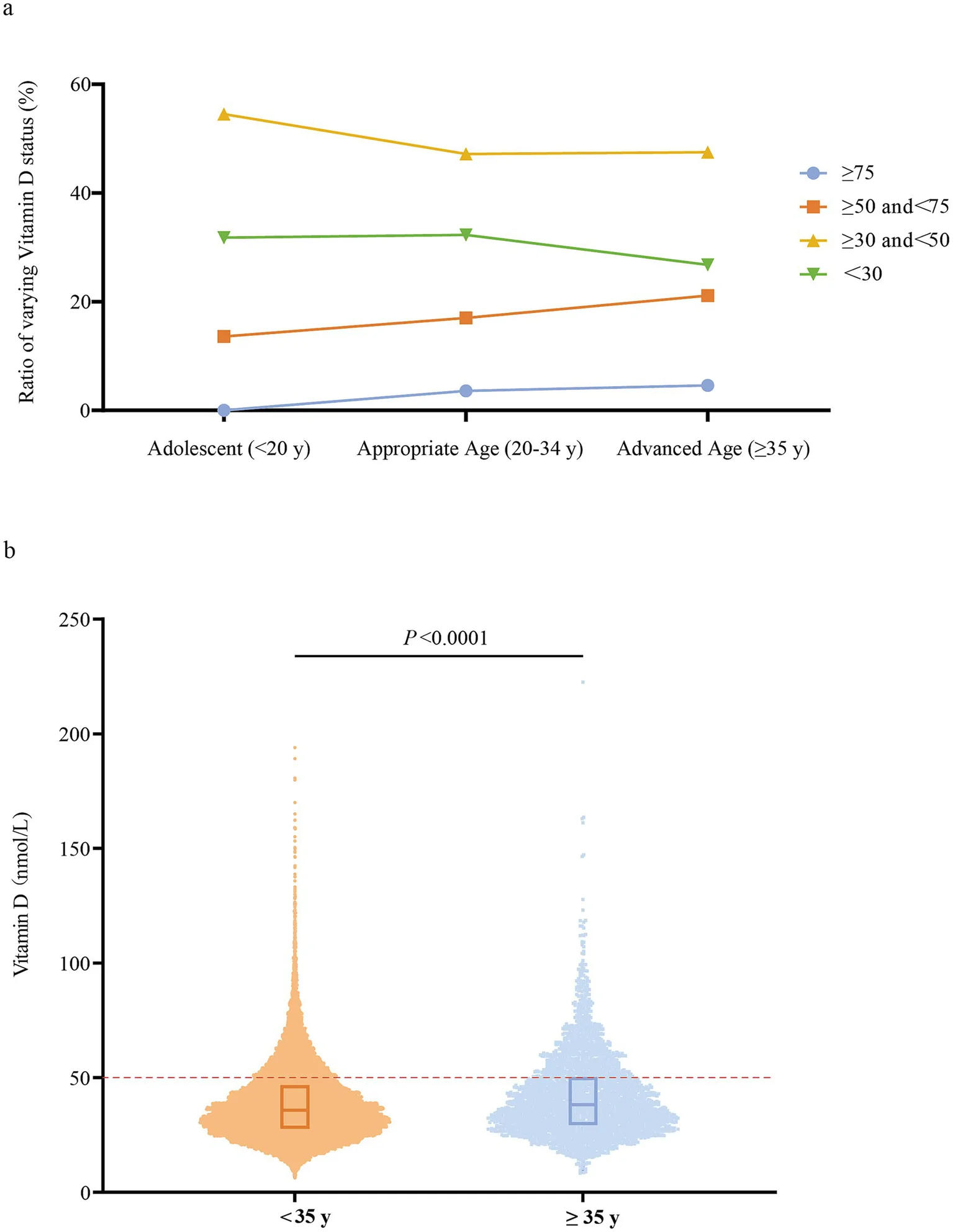
**(a)** The distribution of vitamin D status across different maternal age groups (adolescent, appropriate-age, advanced-age). **(b)** Serum 25(OH)D levels between women aged <35 years and ≥35 years.

Vitamin D levels differed significantly among age groups. The advanced maternal age (AMA) group had the highest median vitamin D level at 38.21 nmol/L. The appropriate-age group had the lowest median level at 35.80 nmol/L. The adolescent group had a small sample size, with a median of 37.07 nmol/L. Using 35 years as the cutoff, pregnant women aged ≥35 years had a significantly higher median vitamin D level than those <35 years; however, the median and third quartile (Q3) for both groups were below 50 nmol/L (Figure 4b and Table 3).

Across all age groups, the proportion of women with deficiency was the highest. The proportion with severe deficiency was highest in the adolescent group and showed a decreasing trend with increasing age. Conversely, the proportion with insufficiency increased with age. The total deficiency rate was 81.0% in the adolescent group, 79.3% in the appropriate-age group, and 74.6% in the AMA group. The combined rate exceeded 96% in all age groups.

### 3.5 Multivariate analysis of factors associated with serum 25(OH)D status

Multinomial logistic regression was performed with vitamin D sufficiency as the reference category. Results are presented in Table 4 and Supplementary Table S2.

**Table 4:** Multinomial logistic regression analysis of factors associated with vitamin D status [ref: ≥75 nmol/L (sufficiency)].

Variable	Severe deficiency aOR (95% CI)	Deficiency aOR (95% CI)	Insufficiency aOR (95% CI)
Age (per year)	**0.90 (0.89–0.92)**	**0.98 (0.96–0.99)**	**0.94 (0.92–0.95)**
Pandemic period			
During (vs. pre)	**0.67 (0.52–0.86)**	**0.55 (0.44–0.69)**	**0.54 (0.43–0.68)**
Post (vs. pre)	**1.59 (1.22–2.07)**	1.04 (0.82–1.33)	0.89 (0.69–1.15)
Season			
Spring (vs. winter)	1.15 (0.92–1.45)	0.96 (0.77–1.20)	0.96 (0.75–1.22)
Summer (vs. winter)	**0.36 (0.29–0.46)**	**0.68 (0.55–0.84)**	1.24 (0.99–1.55)
Autumn (vs. winter)	**0.50 (0.40–0.63)**	**0.74 (0.60–0.92)**	1.14 (0.90–1.44)
Conception method			
Natural (vs. ART)	**2.14 (1.02–4.48)**	**2.21 (1.18–4.11)**	**2.07 (1.04–4.12)**
Number of fetuses			
1 (vs. 3)	1.74 (0.88–3.45)	3.78*E* − 06	4.40*E* − 07
2 (vs. 3)	1.04 (—)	2.52*E* − 06	4.12*E* − 07

^1^Model adjusted for age (continuous), pandemic period, season, conception method, and number of fetuses. ^2^Reference categories: pandemic period: pre-pandemic; season: winter; conception method: ART; number of fetuses: triplets. ^3^Boldness denotes statistical significance (*p* < 0.05).

Season was the strongest associated factor. Compared to winter, summer was associated with significantly lower odds of severe deficiency (aOR = 0.36, 95% CI: 0.29–0.46, *p* < 0.001) and deficiency (aOR = 0.68, 95% CI: 0.55–0.84, *p* < 0.001), but higher odds of insufficiency (aOR = 1.24, 95% CI: 0.99–1.55, *p* = 0.058). Autumn also showed protective associations for severe deficiency (aOR = 0.50, 95% CI: 0.40–0.63, *p* < 0.001) and deficiency (aOR = 0.74, 95%CI: 0.60–0.92, *p* = 0.008). Spring did not show statistically significant differences compared to winter across all categories.

Pandemic period showed significant associations: compared to the pre-pandemic period, the during-pandemic period was associated with lower odds of severe deficiency (aOR = 0.67, 95% CI: 0.52–0.86, *p* = 0.002), deficiency (aOR = 0.55, 95% CI: 0.44–0.69, *p* < 0.001), and insufficiency (aOR = 0.54, 95% CI: 0.43–0.68, *p* < 0.001). However, the post-pandemic period was associated with higher odds of severe deficiency (aOR = 1.59, 95% CI: 1.22–2.07, *p* < 0.001), while the associations for deficiency (aOR = 1.04, 95% CI: 0.82–1.33, *p* = 0.745) and insufficiency (aOR = 0.89, 95% CI: 0.69–1.15, *p* = 0.366) were not statistically significant.

Natural conception was significantly associated with higher odds of severe deficiency (aOR = 2.14, 95% CI: 1.02–4.48, *p* = 0.045), deficiency (aOR = 2.21, 95% CI: 1.18–4.11, *p* = 0.013), and insufficiency (aOR = 2.07, 95% CI: 1.04–4.12, *p* = 0.038) compared to ART conception.

Number of fetuses did not show statistically significant associations with vitamin D status across all categories. Notably, the extreme aOR values (e.g., 3.78*E* − 06) observed for the full sample in the deficiency category were attributable to the small number of triplet pregnancies (*n* = 3), which was resolved after excluding these participants in the sensitivity analysis.

### 3.6 Continuous outcome analysis using GLM univariate and sensitivity analysis

To further examine the factors associated with 25(OH)D levels as a continuous variable, we performed a GLM Univariate analysis with log₁₀-transformed 25(OH)D as the dependent variable. Unlike ordinary linear regression, GLM Univariate correctly handles categorical predictors by generating dummy variables with explicit reference categories, providing unbiased estimates for each factor level.

The model was statistically significant in the full sample (*n* = 20,693: *F* (48, 20,644) = 27.70, *p* < 0.001, *R*^2^ = 0.061; Supplementary Table S3). Maternal age emerged as the strongest predictor (*β* = 0.0047, *p* < 0.001), followed by pandemic period (*F* = 3.82, *p* = 0.022) and season (*F* = 3.34, *p* = 0.018), whereas number of fetuses (*F* = 1.46, *p* = 0.232) and conception method (*F* = 2.15, *p* = 0.142) were not significantly associated. Regression diagnostics were satisfactory: the histogram of standardized residuals approximated a normal distribution (Supplementary Figure S2a), the normal P–P plot showed reasonable alignment along the diagonal (Supplementary Figure S3a), and the scatterplot of standardized residuals versus standardized predicted values displayed random dispersion around zero, indicating homoscedasticity (Supplementary Figure S4a). All variance inflation factors (VIF) were <1.06, ruling out multicollinearity.

Two sensitivity analyses were performed to evaluate the robustness of these findings. First, repeating the GLM Univariate analysis after excluding the three triplet pregnancies (*n* = 20,687) produced virtually identical results (*F* (45, 20,641) = 29.41, *p* < 0.001, *R*^2^ = 0.060; Supplementary Table S4): maternal age remained the strongest predictor (*β* ≈ 0.0047, *p* < 0.001), season and pandemic period maintained borderline significance (*p* ≈ 0.02), while number of fetuses and conception method remained non-significant (*p* > 0.10). Regression diagnostics for this reduced sample were equally satisfactory (Supplementary Figures S2b, S3b, S4b). Second, we excluded six participants (those aged <18 years and those with triplet pregnancies) and repeated the multinomial logistic regression on 20,687 participants. As shown in Supplementary Table S2, all adjusted odds ratios, 95% confidence intervals, and significance levels remained virtually unchanged compared to the full-sample analysis. Notably, the extreme aOR values for number of fetuses observed in the full sample (e.g., singleton versus triplets: aOR = 3.78 × 10^−6^ for the severe deficiency category) were artifacts caused by sparse data (only three triplet pregnancies); after excluding these three participants, the estimates normalized to aOR = 1.50 (95% CI: 0.82–2.75, *p* = 0.189) for the deficiency category and aOR = 1.07 (95% CI: 0.56–2.03, *p* = 0.836) for the insufficiency category, confirming that number of fetuses was not independently associated with vitamin D status. Together, these sensitivity analyses demonstrate that our findings are robust and not driven by the small number of triplet pregnancies or other outliers.

## 4 Discussion

This study is the first to systematically evaluate the vitamin D nutritional status of over 20,000 early-pregnant women in Shanxi Province over a decade (2016–2025). Our findings reveal a persistently high prevalence of vitamin D deficiency, with an overall median serum 25(OH)D level of only 36.12 nmol/L (IQR: 27.87–47.42 nmol/L). A staggering 78.7% of women were deficient, and only 3.7% achieved sufficiency. These results underscore the pervasive and severe nature of vitamin D deficiency in this high-risk northern Chinese population, demanding urgent attention from clinical and public health sectors.

### 4.1 Annual variation and the impact of the pandemic

**Figure 5:**
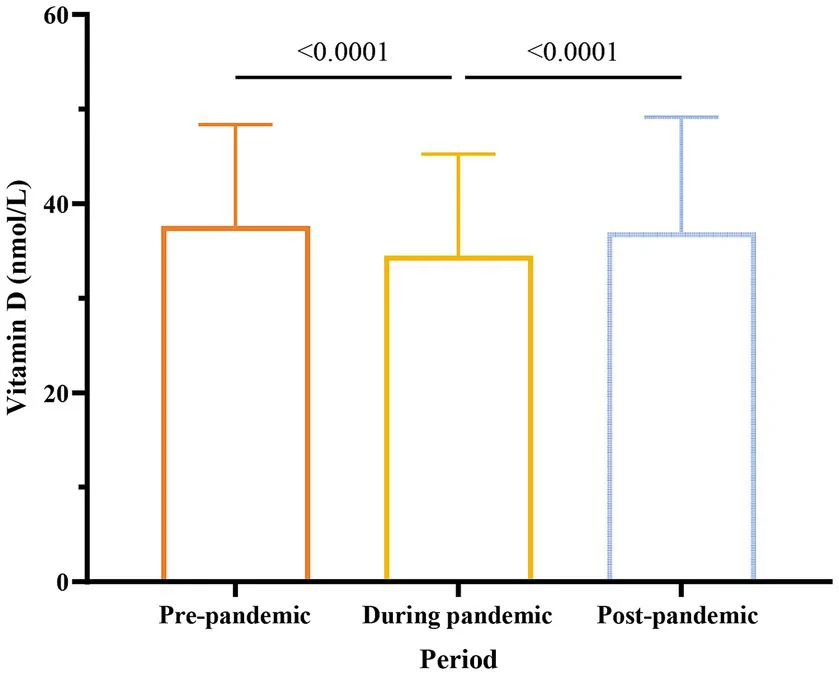
The distribution of vitamin D status across the three periods: pre-pandemic, during pandemic, and post-pandemic.

Longitudinal data revealed that vitamin D levels did not follow a simple linear trend but fluctuated significantly over the decade (Figure 5). During the pre-pandemic period, the proportion of women with severe deficiency showed a promising decline from 48.6% in 2016 to 21.8% in 2019, an improvement that may reflect increasing socioeconomic status and growing awareness of prenatal nutrition. However, this downward trend was abruptly reversed in 2020, when the severe deficiency rate surged to 34.6%, peaking at 37.1% in 2021 and remaining elevated at 35.6% in 2022. The multinomial logistic regression confirmed this shift, showing that the “During pandemic” period was associated with significantly higher odds of severe deficiency compared to the pre-pandemic period. This sharp increase is highly consistent with the implementation of COVID-19 lockdowns and home confinement measures, which drastically reduced outdoor activity and sunlight exposure, a finding supported by numerous global studies (27–31). In the post-pandemic era, while a clear improvement was observed in 2023 and 2024, with the severe deficiency rate dropping to 28.9 and 24.0% respectively, the rate alarmingly rebounded to 34.1% in 2025. This instability suggests that the recovery of vitamin D levels is fragile and may be influenced by other, as-yet-unidentified factors, such as lingering behavioral changes or inconsistent supplementation practices. Despite these fluctuations, the total deficiency rate consistently exceeded 70% every year, and the combined rate of deficiency and insufficiency remained above 95% in most years, highlighting a chronic, severe public health problem.

### 4.2 Seasonal variation

Our study confirmed that season is the strongest environmental determinant of vitamin D status. Serum 25(OH)D levels followed a characteristic pattern: highest in summer (median 40.23 nmol/L) and autumn (37.91 nmol/L), and lowest in spring (33.17 nmol/L) and winter (33.46 nmol/L).

The multinomial logistic regression quantified this relationship. Compared to winter, summer was a powerful protective factor against severe deficiency (aOR = 0.36, 95% CI: 0.29–0.46, *p* < 0.001) and deficiency (aOR = 0.68, 95% CI: 0.55–0.84, *p* < 0.001). Autumn similarly offered significant protection (severe deficiency aOR = 0.50, 95% CI: 0.40–0.63; deficiency aOR = 0.74, 95% CI: 0.60–0.92). Spring, however, did not show a statistically significant difference from winter across all categories, indicating that the risk remains high throughout the colder half of the year.

The clinical impact is stark: the total deficiency rate was highest in spring (88.1%) and winter (82.0%), while the severe deficiency rate in these seasons was 39.7 and 37.7%, respectively. In contrast, summer had the lowest total deficiency rate and the highest sufficiency rate. This seasonal pattern is physiologically driven by the dependence of cutaneous vitamin D synthesis on solar UVB radiation (32). Shanxi Province has a temperate continental monsoon climate, characterized by cold, long winters with weak sunlight intensity and short duration (22), coupled with people wearing covering clothing, leading to a substantial reduction in cutaneous vitamin D synthesis. These findings strongly advocate for targeted vitamin D supplementation strategies, particularly from late autumn through early spring.

### 4.3 Influence of maternal age

Vitamin D levels varied significantly across age groups. The AMA group had the highest median level, while the appropriate-age group had the lowest. The adolescent group, though small in sample size (*n* = 22), exhibited the highest proportion of severe deficiency. Currently, advanced maternal age pregnancy is increasing globally (24, 35). Extensive existing research clearly indicates that advanced maternal age is an associated factor for various adverse pregnancy outcomes and is significantly associated with increased risks of fetal birth defects, especially trisomy 21, cardiovascular malformations, and neurodevelopmental abnormalities (36–40). The logistic regression showed that increasing age (per year) was a significant protective factor against severe deficiency (aOR = 0.90, 95% CI: 0.89–0.92). This counterintuitive finding—that older mothers have better vitamin D status—likely reflects greater health awareness and more proactive engagement in prenatal care. Older pregnant women are generally more diligent about attending consultations, following nutritional advice, and taking supplements, including vitamin D (41). Conversely, the high deficiency rate in the adolescent group highlights a particularly vulnerable subpopulation that requires additional health education and support (33, 34).

### 4.4 Analysis of number of fetuses and conception mode

A notable finding was the significant association between conception mode and vitamin D status. Women who conceived via ART had a higher median vitamin D level than those who conceived naturally. The multivariate analysis confirmed that natural conception was an independent associated factor for all forms of deficiency (severe deficiency aOR = 2.14, 95% CI: 1.02–4.48; deficiency aOR = 2.21, 95% CI: 1.18–4.11). This is plausibly because ART patients undergo rigorous pre-treatment medical evaluations and nutritional optimization, which often includes routine vitamin D screening and supplementation, thereby improving their baseline levels (24–26).

In contrast, the number of fetuses (singleton vs. twins vs. triplets) did not emerge as significant independent factors in the final regression models. While Table 2 shows that women with triplet pregnancies had a notably higher median level, this group was extremely small (*n* = 3). The sensitivity analysis, which excluded participants under 18 and those with triplet pregnancies, confirmed that our primary findings regarding age, pandemic period, season, and conception mode were robust and not driven by these small subgroups.

### 4.5 Comparison on deficiency and insufficiency prevalence among different studies

The results indicate that the overall vitamin D levels in early-pregnant women are severely low, with a median of only 36.12 nmol/L, far below the sufficiency levels recommended by most international guidelines (typically >50 or >75 nmol/L) (42–46). This alarming finding places our cohort among the most severely vitamin D-deficient populations reported globally. A recent meta-analysis by Cristófalo et al. (47) estimated that 68% (95% CI: 60–76%) of pregnant women worldwide have 25(OH)D < 75 nmol/L in the first trimester, underscoring the pervasive nature of this issue.

Within China, marked geographic heterogeneity exists. Studies from eastern coastal cities have documented comparatively better vitamin D status. Ni et al. (48) found that among early-pregnant women in Shanghai, 67.09% were deficient (<50 nmol/L), 29.84% insufficient (50–75 nmol/L), and only 3.07% sufficient (≥75 nmol/L), with a mean level of 43.20 nmol/L—notably higher than our median of 36.12 nmol/L. Cheng et al. (49) reported deficiency and insufficiency rates of 53.1 and 38.5%, respectively, in a Shanghai cohort of 7,816 women, with a mean of 19.6 ng/mL (≈49.0 nmol/L). In southern China (Guangzhou), Yu et al. (50) observed a mean 25(OH)D of 59.3 nmol/L, with only 34.8% deficient and 43.0% insufficient, and found no significant association with preterm birth. In southwestern China (Deyang, Sichuan), Zheng et al. (51) reported a median of 16.3 ng/mL (≈40.8 nmol/L) among 2,742 pregnancies, with 62.5% classified as deficient (<20 ng/mL), and identified early pregnancy (≤12 weeks) as carrying a 3.37-fold higher deficiency risk than late pregnancy. These geographic gradients likely reflect the influence of latitude and sunlight exposure: Shanxi Province (34°–40°N) receives weaker UVB radiation, especially during prolonged winters, compared to lower-latitude regions such as Shanghai (31°N) and Guangzhou (23°N).

Internationally, our results are comparable to those from other high-latitude and culturally conservative regions. In Switzerland, Christoph et al. (52) found that 73.23% of pregnant women had 25(OH)D < 50 nmol/L and 34.2% had severe deficiency (<25 nmol/L), with a mean of 36.72 nmol/L—strikingly similar to our cohort. In Hungary, Polanek et al. (43) reported that only 12.1% of pregnant women reached the optimal level of ≥75 nmol/L. In Canada, Tabatabaei et al. (53) demonstrated that ethnic minority women with 25(OH)D < 75 nmol/L had a 4.05-fold increased risk of preterm birth compared to those with levels ≥75 nmol/L, highlighting the clinical relevance of vitamin D insufficiency. In Australia, Schneuer et al. (54) found that 25(OH)D < 25 nmol/L had limited predictive value for adverse outcomes (AUC = 0.51), suggesting that very low thresholds may be needed to detect risk. In Mexico, Arce-Sánchez et al. (55) reported that 164 of 404 first-trimester women had vitamin D deficiency (<20 ng/mL), which was associated with increased risk of gestational diabetes. In the United States, Beck et al. (14) found that only 20% of first-trimester participants had 25(OH)D < 50 nmol/L, and each 10 nmol/L increase was associated with a 0.05 increase in length-for-age z-score. In Korea, Lee et al. (56) reported that 21.7% were deficient (<10 ng/mL ≈ 25 nmol/L), 41.1% insufficient (10–20 ng/mL), and 37.2% sufficient (≥20 ng/mL), reflecting relatively better status likely due to widespread supplementation practices. In Iran, Abbasalizadeh et al. (57) found a mean serum vitamin D level of 31.77 ng/mL (≈79.4 nmol/L), with 52.8% having hypovitaminosis D, indicating that even in sunny regions, cultural factors such as clothing can limit sun exposure. In Bangladesh, Ahmed et al. (58) reported that 17.3% of pregnant rural women were deficient (<30 nmol/L) and 47.2% insufficient (30–<50 nmol/L), with higher risk among nulliparous and first-trimester women. In Ethiopia, Chane et al. (6) found a 41.41% prevalence of vitamin D deficiency (<20 ng/mL) and 84.11% insufficiency (<30 ng/mL) among 384 pregnant women. In the Middle East, where veiling is common, deficiency rates are particularly high: in Iraq, Mustafa (59) reported that 61.3% of pregnant women had levels <20 ng/mL, 26% insufficient (20–29 ng/mL), and only 12.7% sufficient (30–100 ng/mL). In Lebanon, Ajjour et al. (60) found a mean 25OHD of 15.4 ng/mL (≈38.5 nmol/L) with 72.3% having levels <20 ng/mL, and identified veiling and younger age as significant risk factors. In Jordan, Obeidat et al. (61) reported that summer-born infants had higher cord blood vitamin D levels than winter-born (11.76 vs. 9.39 ng/mL), consistent with our seasonal findings. Collectively, these comparisons underscore that the vitamin D status of early-pregnant women in Shanxi Province ranks among the most severely affected globally, necessitating urgent public health interventions tailored to this high-risk population. The disparities observed across regions can be attributed to differences in latitude and UVB intensity, vitamin D food fortification policies (e.g., mandatory fortification in the United States and Canada), routine prenatal supplementation practices, cultural norms regarding sun exposure and clothing, and socioeconomic factors affecting nutrition and healthcare access.

### 4.6 Guidelines on vitamin D supplementation during early pregnancy

Currently, there is no standardized, unified guidance regarding the dosage of vitamin D supplementation for women in early pregnancy. Based on existing randomized controlled trials and systematic reviews, the World Health Organization (WHO) does not recommend routine vitamin D supplementation for all pregnant women to improve pregnancy outcomes. Instead, it suggests supplementation at approximately 200 IU/day (5 μg/day) or according to national Recommended Nutrient Intakes (RNI) for individuals identified with vitamin D deficiency or at high risk. WHO’s current evidence base considers the effect of vitamin D supplementation on reducing pre-eclampsia, preterm birth, or low birth weight insufficient to support universal intervention globally (62). Similarly, the 2024 Endocrine Society Clinical Practice Guideline notes that while vitamin D has positive effects on pregnant women and fetuses, the optimal dose for empirical supplementation remains unclear due to significant variations in doses used in clinical trials and the allowance for participants to continue personal vitamin D supplementation in many trials (63). In contrast, professional societies in Europe, America, and some high-latitude countries hold more proactive positions. The Institute of Medicine (IOM) and the American College of Obstetricians and Gynecologists (ACOG) recommend a daily intake of 600 IU of vitamin D for pregnant women, noting that supplementation with 1,000–2000 IU/day is considered safe in cases of deficiency, with some studies using up to 4,000 IU/day without observed adverse effects (64, 65). In Asia, the Chinese “Regulation of vitamin D management during peripregnancy period” (2023) recommends 400–800 IU/day for insufficient individuals (20–30 ng/mL); 800–2000 IU/day for deficient individuals (10–20 ng/mL); and for severe deficiency (<10 ng/mL), a higher dose (e.g., 2000 IU/day or more) can be used initially, monitoring levels periodically. After reaching >30 ng/mL, a maintenance dose can be continued if sunlight or dietary intake is limited (66). The 2025 “Consensus on the Clinical Application of Vitamin D and Its Analogs” recommends Chinese pregnant and lactating women supplement 1,200 IU/day (67).

Although the vitamin D status of pregnant women has garnered widespread attention, this study reveals that the overall vitamin D nutritional status of early-pregnant women in Shanxi Province is concerning, with 78.7% being deficient and only 3.7% being in an ideal state (>75 nmol/L). Furthermore, their vitamin D levels are significantly influenced by season, major public events (e.g., the pandemic), age, and the intensity of pre-pregnancy healthcare. The main strength of this study lies in its large sample size and 10 year dynamic observation.

### 4.7 Study limitations

Several limitations of this study should be acknowledged. First, as a hospital-based, single-center retrospective study with restrictive eligibility criteria, our study population may not be fully representative of all early-pregnant women in Shanxi Province, introducing potential selection bias. The participant flow diagram (Supplementary Figure S1) provides transparency regarding the selection process. Second, the Elecsys ECLIA assay used for 25(OH)D measurement is not the gold-standard LC–MS/MS method and may be subject to bias from cross-reactive metabolites (e.g., 3-epi-25-hydroxyvitamin D). However, this potential bias primarily affects the interpretation of absolute prevalence rates, while our findings on relative trends (e.g., seasonal variation) are less impacted. Third, key confounders known to influence vitamin D status—such as individual sun exposure habits, dietary intake, vitamin D supplementation dosage and duration, and pre-pregnancy body mass index—were not available in our retrospective database. Additionally, sociodemographic and lifestyle factors (e.g., education, income, smoking, alcohol consumption, physical activity) were not collected. The inability to adjust for these variables introduces potential residual confounding; therefore, the observed associations should be interpreted cautiously and do not imply causality. Future prospective studies incorporating detailed questionnaires are warranted to validate our findings.

Based on the above findings and limitations, future research should focus on the following aspects: (1) Conducting multi-center, prospective cohort studies combined with detailed dietary, sunlight exposure, and supplement questionnaires to provide a more comprehensive understanding of associated factors; (2) Investigating the cost-effectiveness and feasibility of incorporating vitamin D screening as a routine component of pre-conception or first prenatal visits; (3) Designing and evaluating the effectiveness of personalized vitamin D supplementation regimens tailored to different seasons and risk populations (e.g., adolescents, women conceiving in winter).

## 5 Conclusion

This large-scale retrospective study clearly reveals the severe current situation of widespread vitamin D insufficiency and deficiency among early-pregnant women in Shanxi Province from 2016 to 2025. Seasonal variation is the strongest factor associated with vitamin D levels, with extremely high deficiency rates in winter. Major public health events like the COVID-19 pandemic can exacerbate the risk of vitamin D deficiency. Additionally, older pregnant women and those using assisted reproduction likely received more proactive medical interventions, resulting in relatively better vitamin D status. Therefore, we strongly recommend incorporating the assessment and supplementation of vitamin D levels as a routine and key component of perinatal care. Particularly during winter and for younger pregnant women who may have lower health awareness, universal education and preventive supplementation strategies should be implemented, aiming to fundamentally improve the short- and long-term health outcomes for both mothers and their offspring.

## Data Availability

The original contributions presented in the study are included in the article/Supplementary material, further inquiries can be directed to the corresponding author.

## References

[1] Janoušek J Pilařová V Macáková K Nomura A Veiga-Matos J Silva D . Vitamin D: sources, physiological role, biokinetics, deficiency, therapeutic use, toxicity, and overview of analytical methods for detection of vitamin D and its metabolites. Crit Rev Clin Lab Sci. (2022) 59:517–54. doi: 10.1080/10408363.2022.2070595, 35575431

[2] Samuel S Sitrin M. Vitamin D’s role in cell proliferation and differentiation. Nutr Rev. (2008) 66:S116–24. doi: 10.1111/j.1753-4887.2008.00094.x, 18844838

[3] Artusa P White JH. Vitamin D and its analogs in immune system regulation. Pharmacol Rev. (2025) 77:100032. doi: 10.1016/j.pharmr.2024.100032, 40148037

[4] Holick M Mazzei L Menéndez G Giménez VM Anouti FA Manucha W. Genomic or non-genomic? A question about the pleiotropic roles of vitamin D in inflammatory-based diseases. Nutrients. (2023) 15:767. doi: 10.3390/nu15030767 36771473 PMC9920355

[5] Mavar M Sorić T Bagarić E Sarić A Sarić MM. The power of vitamin D: is the future in precision nutrition through personalized supplementation plans? Nutrients. (2024) 16:1176. doi: 10.3390/nu16081176, 38674867 PMC11054101

[6] Chane E Teketelew B Berta D Walle M Angelo A Cherie N . Prevalence and determinants of vitamin D deficiency among pregnant women in Gondar town 2024: a cross-sectional study from the first and second trimesters. BMJ Open. (2024) 15:e101092. doi: 10.1136/bmjopen-2025-101092, 41130681 PMC12551514

[7] Da Silveira EA Moura L Castro M Kac G Hadler M-C Noll P . Prevalence of vitamin D and calcium deficiency and insufficiency in women of childbearing age and associated risk factors: a systematic review and meta-analysis. Nutrients. (2022) 14:4351. doi: 10.3390/nu14204351, 36297034 PMC9612098

[8] Olstrup H Rylander L Lindh C Malm G Vilhelmsson A. Maternal serum concentrations of vitamin D in pregnancy and preterm birth: a case-control study in southern Sweden. Eur J Nutr. (2025) 64:198. doi: 10.1007/s00394-025-03716-8, 40448825 PMC12126361

[9] Al-Shafei AI Rayis DA Mohieldein AH El-Gendy OA Adam I. Maternal early pregnancy serum level of 25-hydroxyvitamin D and risk of gestational diabetes mellitus. Int J Gynaecol Obstet. (2021) 152:382–5. doi: 10.1002/ijgo.13389, 32976628

[10] Guo H Xie J Yu X Tian Y Guan M Wei J. Effects of vitamin D supplementation on serum 25(OH)D(3) levels and neurobehavioral development in premature infants after birth. Sci Rep. (2024) 14:23972. doi: 10.1038/s41598-024-75191-w, 39397102 PMC11471846

[11] Hart P Lucas R Walsh J Zosky G Whitehouse A Zhu K . Vitamin D in fetal development: findings from a birth cohort study. Pediatrics. (2015) 135:e167–73. doi: 10.1542/peds.2014-1860, 25511121

[12] Svensson N Volqvartz T Vestergaard A Vestergaard E Larsen A Bor P. Effects of maternal vitamin D supplementation on childhood health. Endocr Rev. (2025) 46:922–53. doi: 10.1210/endrev/bnaf034, 41129483 PMC12640715

[13] Zhang Q Yang D Shen Q Li W Li R Tang Y . Correlation of maternal vitamin D status in early pregnancy and vitamin D supplementation during pregnancy with atopic dermatitis in infants: a prospective birth cohort study. Nutrients. (2024) 16:2168. doi: 10.3390/nu16132168, 38999915 PMC11243106

[14] Beck C Blue N Silver R Na M Grobman W Steller J . Maternal vitamin D status, fetal growth patterns, and adverse pregnancy outcomes in a multisite prospective pregnancy cohort. Am J Clin Nutr. (2024) 121:376–84. doi: 10.1016/j.ajcnut.2024.11.018, 39577494 PMC11863332

[15] Fioretto M Barata L Ribeiro I Maciel FA Mattos R De Souza PV . Early and long-term effects of maternal protein restriction on offspring organs and systems: insights from the developmental origins of health and disease (DOHaD). Biogerontology. (2025) 26:175. doi: 10.1007/s10522-025-10316-w, 40877640

[16] Fleming T Watkins A Velazquez M Mathers J Prentice A Stephenson J . Origins of lifetime health around the time of conception: causes and consequences. Lancet. (2018) 391:1842–52. doi: 10.1016/s0140-6736(18)30312-x, 29673874 PMC5975952

[17] Hu Y Wang R Mao D Chen J Li M Li W-D . Vitamin D nutritional status of Chinese pregnant women, comparing the Chinese national nutrition surveillance (CNHS) 2015–2017 with CNHS 2010–2012. Nutrients. (2015) 13:2237. doi: 10.3390/nu13072237, 34209755 PMC8308426

[18] Bai K Dong H Liu L She X Liu C Yu M . Serum 25-hydroxyvitamin D status of a large Chinese population from 30 provinces by LC–MS/MS measurement for consecutive 3 years: differences by age, sex, season and province. Eur J Nutr. (2023) 62:1503–16. doi: 10.1007/s00394-023-03094-z, 36692589

[19] Zhang Q Zhang C Wang Y Zhao J Li H Shen Q . Relationship of maternal obesity and vitamin D concentrations with fetal growth in early pregnancy. Eur J Nutr. (2021) 61:915–24. doi: 10.1007/s00394-021-02695-w, 34657185 PMC8854300

[20] CarusoR Di MuzioM Di SimoneE DionisiS MagonA ConteG . Retrospective analysis of preventable procedural adverse events (ICD-10 Y62-Y69) in the TriNetX network: a multiregional study before, during and after the COVID-19 pandemic. BMJ Qual Saf. (2025). doi: 10.1136/bmjqs-2025-019077, 40784756

[21] Tagini F Uldum SA Berengua C Ivan B Capaul R Edouard S . Epidemiological changes in *Chlamydia pneumoniae* molecular detections before, during and after the COVID-19 pandemic in 27 European sites and Taiwan, 2018 to 2023. Euro Surveill. (2025) 30:2400682. doi: 10.2807/1560-7917.ES.2025.30.23.2400682, 40511472 PMC12164279

[22] National Climate Center (2024) China climate bulletin. Available online at: https://www.ncc-cma.net/channel/news/newsid/100659

[23] Chen Y Kong G. Changes in vitamin D status among adults from the COVID-19 pandemic to post-pandemic normality. Front Nutr. (2024) 11:1407890. doi: 10.3389/fnut.2024.1407890, 39155929 PMC11327124

[24] Kooij I Holt R Juel Mortensen L Lorenzen M Bentin-Ley U Krog H . Follicular fluid concentrations of the vitamin D metabolite 24,25(OH)2D3 are positively associated with live birth rate after assisted reproductive technology. J Steroid Biochem Mol Biol. (2025) 251:106764. doi: 10.1016/j.jsbmb.2025.106764, 40245991

[25] Baldini GM Russo M Proietti S Forte G Baldini D Trojano G. Supplementation with vitamin D improves the embryo quality in in vitro fertilization (IVF) programs, independently of the patients’ basal vitamin D status. Arch Gynecol Obstet. (2024) 309:2881–90. doi: 10.1007/s00404-024-07473-7, 38580857 PMC11147876

[26] Antunes RA Melo BML Souza M Souza MM Melo GPS Jandre TFM . Vitamin D and follicular recruitment in the in vitro fertilization cycle. JBRA Assist Reprod. (2024) 28:269–75. doi: 10.5935/1518-0557.20240005, 38381779 PMC11152436

[27] Ramírez-Stieben LA Nollas F Gloria S Belardinelli MV Pustilnik E Bolzán D . 25(OH)D levels during the COVID-19 pandemic: impact of lockdown and ultraviolet radiation. Gac Med Mex. (2023) 159:185–93. doi: 10.24875/GMM.M23000770, 37494711

[28] Agüero-Domenech N Bernabeu E García-Valentín A Sarrión A Jover S Baranda J . Influence of strict lockdown on vitamin D deficiency in pregnant women: a word of caution. Nutrients. (2023) 15:1972. doi: 10.3390/nu15081972, 37111192 PMC10143695

[29] Szarpak L Feduniw S Pruc M Ciebiera M Cander B Rahnama-Hezavah M . The vitamin D serum levels in pregnant women affected by COVID-19: a systematic review and meta-analysis. Nutrients. (2023) 15:2588. doi: 10.3390/nu15112588, 37299555 PMC10255765

[30] Vásquez-Procopio J Torres-Torres J Borboa-Olivares H Sosa SEY Martínez-Portilla RJ Solis-Paredes M . Association between 25-OH vitamin D deficiency and COVID-19 severity in pregnant women. Int J Mol Sci. (2022) 23:15188. doi: 10.3390/ijms232315188 36499537 PMC9735729

[31] Knuschke P. Sun exposure and vitamin D. Curr Probl Dermatol. (2021) 55:296–315. doi: 10.1159/000517640, 34698034

[32] Li H Ma J Huang R Wen Y Liu G Xuan M . Prevalence of vitamin D deficiency in the pregnant women: an observational study in Shanghai, China. Arch Public Health. (2020) 78:31. doi: 10.1186/s13690-020-00414-1 32518650 PMC7271532

[33] Öcal DF Aycan Z Dağdeviren G Kanbur N Küçüközkan T Derman O. Vitamin D deficiency in adolescent pregnancy and obstetric outcomes. Taiwan J Obstet Gynecol. (2019) 58:778–83. doi: 10.1016/j.tjog.2019.09.008, 31759526

[34] Keats EC Akseer N Thurairajah P Cousens S Bhutta ZA. Multiple-micronutrient supplementation in pregnant adolescents in low- and middle-income countries: a systematic review and a meta-analysis of individual participant data. Nutr Rev. (2022) 80:141–56. doi: 10.1093/nutrit/nuab004, 33846729 PMC8754251

[35] Wang K Dong F Ma S Bu Z. The association between vitamin D deficiency and clinical pregnancy rate in IVF patients with different age. Front Endocrinol. (2024) 15:1485238. doi: 10.3389/fendo.2024.1485238, 39829955 PMC11738904

[36] Elmerdahl Frederiksen L Ølgaard SM Roos L Petersen OB Rode L Hartwig T . Maternal age and the risk of fetal aneuploidy: a nationwide cohort study of more than 500,000 singleton pregnancies in Denmark from 2008 to 2017. Acta Obstet Gynecol Scand. (2024) 103:351–9. doi: 10.1111/aogs.14713, 37986093 PMC10823394

[37] Mamasoula C Bigirumurame T Chadwick T Addor MC Cavero-Carbonell C Dias CM . Maternal age and the prevalence of congenital heart defects in Europe, 1995–2015: a register-based study. Birth Defects Res. (2023) 115:583–94. doi: 10.1002/bdr2.2152, 36734416

[38] Khalilipalandi S Lemieux A Lauzon-Schnittka J Perreault L Dubois M Tousignant A . Systematic review and meta-analysis of prenatal risk factors for congenital heart disease: part 1, maternal chronic diseases and parental exposures. Can J Cardiol. (2024) 40:2476–95. doi: 10.1016/j.cjca.2024.07.004, 38996968

[39] Ziętek MM Jaszczyk A Stankiewicz AM Sampino S. Prenatal gene-environment interactions mediate the impact of advanced maternal age on mouse offspring behavior. Sci Rep. (2024) 14:31733. doi: 10.1038/s41598-024-82070-x, 39738558 PMC11685589

[40] Gao L Li S Yue Y Long G. Maternal age at childbirth and the risk of attention-deficit/hyperactivity disorder and learning disability in offspring. Front Public Health. (2023) 11:923133. doi: 10.3389/fpubh.2023.923133, 36817892 PMC9931903

[41] Lu Y Zhang X Wu S Zhang S Tan J. A bibliometric analysis of global research on vitamin D and reproductive health between 2012 and 2021: learning from the past, planning for the future. Front Nutr. (2022) 9:973332. doi: 10.3389/fnut.2022.973332, 36159484 PMC9493010

[42] Gjerde J Kjellevold M Dahl L Berg T Bøkevoll A Markhus M. Validation and determination of 25(OH) vitamin D and 3-Epi25(OH)D3 in breastmilk and maternal- and infant plasma during breastfeeding. Nutrients. (2020) 12:2271. doi: 10.3390/nu12082271, 32751196 PMC7469027

[43] Polanek E Sisák A Molnár R Máté Z Horváth E Németh G . A study of vitamin D status and its influencing factors among pregnant women in Szeged, Hungary: a secondary outcome of a case–control study. Nutrients. (2024) 16:1431. doi: 10.3390/nu16101431, 38794669 PMC11123871

[44] Saluja S Sugathan N Krishnamurthy R Jude E. Impact of vitamin D deficiency on gestational diabetes and pregnancy outcomes across diverse ethnic groups: a retrospective cohort study. Nutrients. (2025) 17:565. doi: 10.3390/nu17030565, 39940423 PMC11820082

[45] Dunlop E Pham NM Van Hoang D Mazahery H Neo B Shrapnel J . A systematic review and meta-analysis of circulating 25-hydroxyvitamin D concentration and vitamin D status worldwide. J Public Health. (2025) 47:e520–9. doi: 10.1093/pubmed/fdaf080, 40652566 PMC12670000

[46] Liu W Hu J Fang Y Wang P Lu Y Shen N. Vitamin D status in mainland of China: a systematic review and meta-analysis. EClinicalMedicine. (2021) 38:101017. doi: 10.1016/j.eclinm.2021.101017, 34308318 PMC8283334

[47] Cristófalo MM de Almeida Garcia JO Aldrighi JFS Cristófalo RM França MLM Luzia LA . Prevalence of vitamin D deficiency in pregnant women: systematic review and meta-analysis. Nutr Rev. (2026) 84:600–14. doi: 10.1093/nutrit/nuaf168, 40973701

[48] Ni M Zhang Q Zhao J Shen Q Yao D Wang T . Relationship between maternal vitamin D status in the first trimester of pregnancy and maternal and neonatal outcomes: a retrospective single center study. BMC Pediatr. (2021) 21:330. doi: 10.1186/s12887-021-02730-z, 34325665 PMC8320191

[49] Cheng Y Chen J Li T Pei J Fan Y He M . Maternal vitamin D status in early pregnancy and its association with gestational diabetes mellitus in Shanghai: a retrospective cohort study. BMC Pregnancy Childbirth. (2022) 22:819. doi: 10.1186/s12884-022-05149-1, 36335302 PMC9636619

[50] Yu L Guo Y Ke HJ He YS Che D Wu JL. Vitamin D status in pregnant women in southern China and risk of preterm birth: a large-scale retrospective cohort study. Med Sci Monit. (2019) 25:7755–62. doi: 10.12659/MSM.919307, 31617502 PMC6816329

[51] Zheng M Wang Y Zou N Jianfei E Zou Y. Vitamin D deficiency and pregnancy outcomes: LC-MS/MS-based evaluation in southwest China. Front Med. (2026) 12:1711506. doi: 10.3389/fmed.2025.1711506 PMC12872777 41657570

[52] Christoph P Challande P Raio L Surbek D. High prevalence of severe vitamin D deficiency during the first trimester in pregnant women in Switzerland and its potential contributions to adverse outcomes in the pregnancy. Swiss Med Wkly. (2020) 150:w20238. doi: 10.4414/smw.2020.20238, 32502277

[53] Tabatabaei N Auger N Herba CM Wei S Allard C Fink GD . Maternal vitamin D insufficiency early in pregnancy is associated with increased risk of preterm birth in ethnic minority women in Canada. J Nutr. (2017) 147:1145–51. doi: 10.3945/jn.116.241216, 28424259

[54] Schneuer FJ Roberts CL Guilbert C Simpson JM Algert CS Khambalia AZ . Effects of maternal serum 25-hydroxyvitamin D concentrations in the first trimester on subsequent pregnancy outcomes in an Australian population. Am J Clin Nutr. (2014) 99:287–95. doi: 10.3945/ajcn.113.065672, 24257720

[55] Arce-Sánchez L González-Ludlow I Lizano-Jubert I Almada-Balderrama JA Suárez-Rico BV Montoya-Estrada A . First trimester vitamin D deficiency and risk of gestational diabetes mellitus in a Mexican cohort. Nutrients. (2026) 18:97. doi: 10.3390/nu18010097, 41515214 PMC12787668

[56] Lee JY Jung SH Ahn EH Ryu HM. Assessing the influence of maternal vitamin D deficiency in early pregnancy and subsequent improvement on perinatal outcomes and long-term child development: a retrospective cohort study. PLoS One. (2025) 20:e0323146. doi: 10.1371/journal.pone.0323146, 40445942 PMC12124564

[57] Abbasalizadeh F Bavand S Fathnezhad-Kazemi A. Prevalence and determinants of hypovitaminosis D in early pregnancy: a cross-sectional study among first-trimester pregnant women. BMC Pregnancy Childbirth. (2025) 25:1028. doi: 10.1186/s12884-025-08233-4, 41053671 PMC12502393

[58] Ahmed F Khosravi-Boroujeni H Khan MR Roy AK Raqib R. Prevalence and predictors of vitamin D deficiency and insufficiency among pregnant rural women in Bangladesh. Nutrients. (2021) 13:449. doi: 10.3390/nu13020449, 33572898 PMC7911263

[59] Mustafa A. Assessment of vitamin D, vitamin B12, and folate levels in recently identified pregnant females. Cureus. (2024) 16:e68514. doi: 10.7759/cureus.68514, 39364513 PMC11447766

[60] Ajjour S Chakhtoura M Nassar A Assaad M Rahme M Cooper C . Vitamin D insufficiency in pregnant women from Lebanon: prevalence and key predictors. Reprod Health. (2025) 22:158. doi: 10.1186/s12978-025-02028-8, 40903752 PMC12406351

[61] Obeidat RA Sakee B Alguzo S Alshwayyat S Altal OF Rawashdeh H. Vitamin D status at birth among pregnant women and their newborns in Jordan: a cross-sectional study. Front Med. (2026) 13:1864210. doi: 10.3389/fmed.2026.1864210 PMC13286968 42344505

[62] WHO. Antenatal Care Recommendations for a Positive Pregnancy Experience: Nutritional Interventions Update: Vitamin D Supplements during Pregnancy. Geneva: WHO (2020). 32783436

[63] Demay MB Pittas AG Bikle DD Diab DL Kiely ME Lazaretti-Castro M . Vitamin D for the prevention of disease: an endocrine society clinical practice guideline. J Clin Endocrinol Metab. (2024) 109:1907–47. doi: 10.1210/clinem/dgae290, 38828931

[64] Eleanor Dunlop Ngoc Minh Pham Dong Van Hoang Hajar Mazahery Belinda Neo Jillian Shrapnel . ACOG Committee opinion no. 495: vitamin D: screening and supplementation during pregnancy. Obstet Gynecol. (2011) 118:197–8. doi: 10.1097/AOG.0b013e318227f06b 21691184

[65] Pérez-López FR Pilz S Chedraui P. Vitamin D supplementation during pregnancy: an overview. Curr Opin Obstet Gynecol. (2020) 32:316–21. doi: 10.1097/GCO.0000000000000641, 32487800

[66] China Medical Education Association. Regulation of Vitamin D Management during Peripregnancy Period. Beijing: China Medical Education Association (2023).

[67] Chinese Society of Osteoporosis and Bone Mineral Research, Chinese Medical Association. Consensus on the clinical application of vitamin D and its analogues (2025). Chin J Osteoporos Bone Miner Dis. (2025) 18:497–517. doi: 10.3969/j.issn.1674-2591.2025.05.001

