# Supplementary material for: A decade-long assessment reveals persistent vitamin D deficiency and its associated factors in early-pregnant women from northern China (2016–2025)
Source: Front Nutr. 2026 Aug 25;13:1881959. doi: 10.3389/fnut.2026.1881959 (PMC13549878; doi:10.3389/fnut.2026.1881959)
Supplement: Supplementary file 1 [file Supplementary_file_1.docx]

**Table S1** Annual distribution of vitamin D status categories (2016-2025).

|  | **Total n**  **(%)** | **2016** | **2017** | **2018** | **2019** | **2020** | **2021** | **2022** | **2023** | **2024** | **2025** |
| --- | --- | --- | --- | --- | --- | --- | --- | --- | --- | --- | --- |
| **Vitamin D status** |  | | | | | | | | | | |
| **Sufficiency** | 771  (3.7%) | 0  (0.0%) | 19  (2.3%) | 36  (3.1%) | 41  (3.1%) | 24  (1.7%) | 83  (2.1%) | 116  (3.8%) | 119  (4.1%) | 166  (5.3%) | 167  (6.0%) |
| **Insufficiency** | 3,639  (17.6%) | 10  (13.5%) | 138  (16.5%) | 248  (21.5%) | 264  (20.1%) | 227  (16.0%) | 582  (14.4%) | 535  (17.6%) | 509  (17.7%) | 616  (19.5%) | 510  (18.3%) |
| **Deficiency** | 9,779  (47.3%) | 28  (37.8%) | 389  (46.4%) | 590  (51.2%) | 724  (55.0%) | 675  (47.7%) | 1,872  (46.4%) | 1,310  (43.0%) | 1,420  (49.3%) | 1,615  (51.2%) | 1,156  (41.5%) |
| **Severe deficiency** | 6,504  (31.4%) | 36  (48.6%) | 292  (34.8%) | 279  (24.2%) | 287  (21.8%) | 489  (34.6%) | 1,495  (37.1%) | 1,083  (35.6%) | 834  (28.9%) | 759  (24.0%) | 950  (34.1%) |

**Table S2.** Comparison of multinomial logistic regression results between full sample (n=20,693) and sample after excluding participants aged <18 years and triplet pregnancies (n=20,687)

| **Vitamin D status** | **Full sample (n=20,693)**  **aOR (95% CI)** | **After exclusion (n=20,687)**  **aOR (95% CI)** |
| --- | --- | --- |
| **Insufficiency** |  |  |
| Age | 0.98 (0.96–0.99) | 0.98 (0.96–0.99) |
| Post-pandemic vs Pre-pandemic | 0.54 (0.43–0.68) | 0.54 (0.43–0.69) |
| During pandemic vs Pre-pandemic | 0.89 (0.69–1.15) | 0.89 (0.69–1.16) |
| Season （Ref：Winter） |  |  |
| Spring | 0.96 (0.75–1.22) | 0.95 (0.75–1.22) |
| Summer | 1.24 (0.99–1.55) | 1.23 (0.99–1.55) |
| Autumn | 1.14 (0.90–1.44) | 1.14 (0.90–1.43) |
|  |  |  |
| Conception (Natural vs ART) | 2.07 (1.04–4.12) | 2.07 (1.04–4.12) |
| Number of fetuses (Twins vs Singleton) | 0.94 (0.49-1.78) | 0.94 (0.49-1.78) |
| Number of fetuses (Triplets vs Singleton) | 337090876.83 (29770797.51-3816836253.49) | — |
|  |  |  |
| **Deficiency** |  |  |
| Age | 0.94 (0.92–0.95) | 0.94 (0.92–0.95) |
| Post-pandemic vs Pre-pandemic | 0.55 (0.44–0.69) | 0.55 (0.44–0.69) |
| During pandemic vs Pre-pandemic | 1.04 (0.82–1.33) | 1.04 (0.82–1.33) |
| Season （Ref：Winter） |  |  |
| Spring | 0.96 (0.77–1.20) | 0.96 (0.77–1.20) |
| Summer | 0.68 (0.55–0.84) | 0.68 (0.55–0.84) |
| Autumn | 0.74 (0.60–0.92) | 0.74 (0.60–0.92) |
|  |  |  |
| Conception (Natural vs ART) | 2.21 (1.18–4.11) | 2.21 (1.18–4.11) |
| Number of fetuses (Twins vs Singleton) | 0.67 (0.36–1.22) | 0.67 (0.36–1.22) |
| Number of fetuses (Triplets vs Singleton) | 39220102.45 (39220102.45-39220102.45) | — |
|  |  |  |
| **Severe deficiency** |  |  |
| Age | 0.90 (0.89–0.92) | 0.90 (0.89–0.92) |
| Post-pandemic vs Pre-pandemic | 0.67 (0.53–0.86) | 0.67 (0.52–0.86) |
| During pandemic vs Pre-pandemic | 1.59 (1.23–2.07) | 1.59 (1.22–2.07) |
| Season （Ref：Winter） |  |  |
| Spring | 1.15 (0.92–1.45) | 1.15 (0.92–1.45) |
| Summer | 0.36 (0.29–0.46) | 0.36 (0.29–0.46) |
| Autumn | 0.50 (0.40–0.63) | 0.50 (0.40–0.63) |
|  |  |  |
| Conception (Natural vs ART) | 2.14 (1.02–4.48) | 2.13 (1.02–4.48) |
| Number of fetuses (Twins vs Singleton) | 0.60 (0.30–1.18) | 0.60 (0.30–1.18) |
| Number of fetuses (Triplets vs Singleton) | 0.57 (—) | — |

**Table S3. General Linear Model (GLM) Univariate analysis of log_10_-transformed 25(OH)D levels in the full sample (n=20,693).**

| **Variable** | **β** | **SE** | **t** | **p** |
| --- | --- | --- | --- | --- |
| Intercept | 1.323048 | 0.076364 | 17.325 | <0.001 |
| Age | 0.004728 | 0.000301 | 15.729 | <0.001 |
| **Season** *(ref: Winter)* | F=3.34, p=0.018* | | | |
| Spring vs Winter | -0.034911 | 0.107189 | -0.326 | 0.745 |
| Summer vs Winter | 0.114702 | 0.102625 | 1.118 | 0.264 |
| Autumn vs Winter | 0.113984 | 0.102626 | 1.111 | 0.267 |
|  |  |  |  |  |
| **Number of fetuses** *(ref: Singleton)* | F=1.46, p=0.232 | | | |
| Triplet vs Singleton | 0.091057 | 0.169524 | 0.537 | 0.591 |
| Twin vs Singleton | 0.245660 | 0.185660 | 1.323 | 0.186 |
|  |  |  |  |  |
| **Conception** *(ref: IVF)* | F=2.15, p=0.142 | | | |
| Natural vs IVF | 0.071740 | 0.076034 | 0.944 | 0.345 |
|  |  |  |  |  |
| **Pandemic** *(ref: Pre-pandemic)* | F=3.82, p=0.022* | | | |
| During vs Pre-pandemic | 0.058406 | 0.087524 | 0.667 | 0.505 |
| Post vs Pre-pandemic | 0.188585 | 0.102629 | 1.838 | 0.066 |
| *Model: R² = 0.061, Adj R² = 0.058, F(48, 20644) = 27.70, p<0.001* | | | | |

**Table S4. General Linear Model (GLM) Univariate analysis of log_10_-transformed 25(OH)D levels after excluding triplet pregnancies (n=20,687).**

| **Variable** | **β** | **SE** | **t** | **p** |
| --- | --- | --- | --- | --- |
| Intercept | 1.323048 | 0.076364 | 17.325 | <0.001 |
| Age | 0.004728 | 0.000301 | 15.729 | <0.001 |
| **Season** *(ref: Winter)* | F=3.276, p=0.02* | | | |
| Spring vs Winter | 0.149409 | 0.062863 | 2.377 | 0.017 |
| Summer vs Winter | 0.148881 | 0.062866 | 2.368 | 0.018 |
| Autumn vs Winter | 0.034893 | 0.107188 | 0.326 | 0.745 |
|  |  |  |  |  |
| **Number of fetuses** *(ref: Singleton)* | F=0.11, p=0.739 | | | |
| Twin vs Singleton | 0.245600 | 0.185658 | 1.323 | 0.186 |
|  |  |  |  |  |
| **Conception** *(ref: IVF)* | F=2.16, p=0.142 | | | |
| Natural vs IVF | -0.071723 | 0.076033 | -0.943 | 0.346 |
|  |  |  |  |  |
| **Pandemic** *(ref: Pre-pandemic)* | F=3.83, p=0.022* | | | |
| During vs Pre-pandemic | 0.058406 | 0.087524 | 0.667 | 0.505 |
| Post vs Pre-pandemic | 0.188585 | 0.102629 | 1.838 | 0.066 |
| *Model: R² = 0.060, Adj R² = 0.058, F(45, 20641) = 29.41, p<0.001* | | | | |

**Figure S1**

Baseline characteristics of early-pregnant women in Shanxi Province (2016–2025).

**Figure S2 Histogram of standardized residuals**

(a) Before exclusion (n=20,693); (b) After exclusion (n=20,687)

**a**

**b**

**Table S3 Normal P-P plot of standardized residuals**

(a)Before exclusion (n=20,693); (b) After exclusion (n=20,687)

**a**

**b**

**Figure S4 Scatterplot of standardized residuals vs standardized predicted values**

(a) Before exclusion (n=20,693); (b) After exclusion (n=20,687)

**a**

**b**
